# Gene–gene interaction analysis for the survival phenotype based on the Cox model

**DOI:** 10.1093/bioinformatics/bts415

**Published:** 2012-09-03

**Authors:** Seungyeoun Lee, Min-Seok Kwon, Jung Mi Oh, Taesung Park

**Affiliations:** ^1^Department of Mathematics and Statistics, Sejong University, Seoul 143-747; ^2^Interdisciplinary Program in Bioinformatics; ^3^College of Pharmacy and Research Institute of Pharmaceutical Sciences; ^4^Department of Statistics, Seoul National University, Seoul 151-747, Korea

## Abstract

**Motivation:** For the past few decades, many statistical methods in genome-wide association studies (GWAS) have been developed to identify SNP–SNP interactions for case-control studies. However, there has been less work for prospective cohort studies, involving the survival time. Recently, Gui *et al.* (2011) proposed a novel method, called Surv-MDR, for detecting gene–gene interactions associated with survival time. Surv-MDR is an extension of the multifactor dimensionality reduction (MDR) method to the survival phenotype by using the log-rank test for defining a binary attribute. However, the Surv-MDR method has some drawbacks in the sense that it needs more intensive computations and does not allow for a covariate adjustment. In this article, we propose a new approach, called Cox-MDR, which is an extension of the generalized multifactor dimensionality reduction (GMDR) to the survival phenotype by using a martingale residual as a score to classify multi-level genotypes as high- and low-risk groups. The advantages of Cox-MDR over Surv-MDR are to allow for the effects of discrete and quantitative covariates in the frame of Cox regression model and to require less computation than Surv-MDR.

**Results:** Through simulation studies, we compared the power of Cox-MDR with those of Surv-MDR and Cox regression model for various heritability and minor allele frequency combinations without and with adjusting for covariate. We found that Cox-MDR and Cox regression model perform better than Surv-MDR for low minor allele frequency of 0.2, but Surv-MDR has high power for minor allele frequency of 0.4. However, when the effect of covariate is adjusted for, Cox-MDR and Cox regression model perform much better than Surv-MDR. We also compared the performance of Cox-MDR and Surv-MDR for a real data of leukemia patients to detect the gene–gene interactions with the survival time.

**Contact:** leesy@sejong.ac.kr; tspark@snu.ac.kr

## 1 INTRODUCTION

Recently, massive amounts of information for single-nucleotide polymorphisms (SNPs) across the whole genome have become available from high-throughput technology, which allows genome-wide association studies (GWAS) to be performed. As recently reviewed by Manolio (2010), nearly 600 genome-wide association studies covering 150 distinct diseases and traits have been reported, with nearly 800 SNP-trait associations reported as significant under *P <* 5 × 10^−8^. However, it is noted that the effective sizes of the loci identified via GWAS are relatively small, and these individual loci may not be useful in assessing risk in personal genetics, as mentioned by Moore and Williams (2009).

In early GWAS, statistical methods for identifying susceptibility have considered a single SNP at a time and have selected a subset of the top few SNPs from a ranked list of SNPs. Then, replication studies have been implemented to determine whether these associations held for other samples. Some of the replication studies, however, show that significant associations are not found from the top ranked list. Recently, this single-locus approach has been moved into a multiple-loci approach because most complex diseases are associated with multiple genes and their interactions. However, the traditional parametric approach, such as the logistic regression model, has limited power in detecting non-linear patterns of interaction and needs a large amount of study samples when multiple SNPs and gene–gene interactions are considered.

As a dimensional reduction strategy, Ritchie *et al*. (2001) proposed the multifactor dimensionality reduction (MDR) method, which is a computationally efficient method for detecting non-linear patterns of gene–gene interactions in genetic association studies. The MDR method is a non-parametric and genetic model-free approach that efficiently identifies higher-order interactions between genes and/or gene–environmental factors with binary phenotype. The main idea of MDR is to reduce multi-dimensional genotypes into one-dimensional binary attributes by pooling genotypes of multiple SNPs using a well-defined classifier. More studies on MDR have been published by Hahn *et al*. (2003), Moore (2004) and Hahn and Moore (2004). In addition, many modifications and extensions to MDR have been published, which include the use of odds ratios (Chung *et al*., 2007), log-linear models (Lee *et al*., 2007), generalized linear models (Lou *et al*., 2007), methods for imbalanced data (Velez *et al*., 2007), methods for dealing with missing data (Namkung *et al*., 2009), and model-based methods (Calle *et al*., 2008). Among these previous studies, the generalized multifactor dimensionality reduction (GMDR) method proposed by Lou *et al*. (2007) includes both dichotomous and continuous phenotypes and allows for the adjustment of covariates such as age, sex and other clinical variables.

In a prospective cohort study, survival time has been one of the important phenotypes in studies of associations with gene expression levels measured by high-throughput microarray technology. Recently, Gui *et al*. (2010) proposed a novel approach for identifying gene–gene interactions with survival times using SNP information in the frame of MDR, called Surv-MDR. The Surv-MDR method modifies MDR's constructive induction algorithm to classify multi-level genotypes as high- and low-risk groups using a log-rank test instead of case control ratios. In addition, balanced accuracy is replaced by log-rank test statistics and is used as a score to determine the best model. Surv-MDR was shown to have better performance than that of traditional Cox regression models through simulation experiments and was successfully applied to the identification of SNP–SNP interactions associated with survival time in bladder cancer data (Andrew *et al*., 2009).

Although Surv-MDR was shown to be powerful in gene–gene interaction analysis for survival times, Surv-MDR has major drawbacks in its application to GWAS. First, Surv-MDR requires very intensive computations by computing log-rank test statistics for all possible combinations of SNPs. Second, Surv-MDR cannot allow for covariate adjustment, although adjustment of individual-specific covariates is very important in association studies because the true genetic associations with the survival phenotype may be confounded by the covariates such as age, sex, race and stage.

To overcome these drawbacks, we propose a new approach, called the Cox-MDR method, which is an extension of GMDR to the survival time using the martingale residual as a score obtained from a Cox model. The Cox model has been the most widely used to access the effects of risk factors on survival times since a proportional hazards assumption was proposed in the framework of the regression model by Cox (1972). In the Cox model, the effect of covariates is multiplicative with the hazard rate and is easily estimated without any consideration of the hazard function if the proportional hazards assumption holds. Since the martingale residual is difference between the counting process and the integrated intensity function in the Cox model, it can be intuitively interpreted as the excess deaths (Therneau *et al*., 1990). The Cox-MDR uses this martingale residual to identify the association between potential genetic factors and the survival time. The martingale residual of each individual is obtained from the reduced model with no SNP effect and is used as a new classifier of high- and low-risk groups whereas all of the other MDR procedures are kept unchanged. The effects of covariates are adjusted in the reduced Cox model from which the martingale residual is produced.

We compare the power of Cox-MDR with those of Surv-MD and Cox regression model through simulation studies with 40 different penetrance models listed by Velez *et al*. (2007). These 40 models were constructed under combinations of four different heritability and two different minor allele frequencies. The power comparison is made under without and with adjusting for covariates.

## 2 METHOD: COX-MDR

In this section, we introduce a Cox model and describe how Cox-MDR is constructed by incorporating the martingale residual into the frame of GMDR.

Let T_i_* and C_i_* denote the survival time and censoring time for the *i*th individual, respectively. Let *T_i_* = min(T_i_*,C_i_*) be the observed time and let *δ_i_* = *I*(T_i_* ⩽ C_i_*) be an indicator for uncensored observation. The observed data consists of (*T_i_*, *δ_i_*) pairs as well as covariates that may be vector-valued and/or time-varying, notated as *X_i_*(*t*). Here we consider only time-fixed covariates, notated as *X_i_*. The counting process formulation replaces the pair of variables (*T_i_*, *δ_i_*) with the pair of functions (*N_i_*(*t*),*Y_i_*(*t*)), where *N_i_*(*t*) = *I*(*T_i_*⩽*t*, *δ_i_*= 1) and *Y_i_*(*t*) = *I*(*T_i_*⩾*t*). Here, *N_i_*(*t*) is a counting process that represents the number of observed events by time *t* for the *i*th individual and *Y_i_*(*t*) is a predictable process that represents the risk set at time *t*.

The Cox model assumes a hazard function of the following form

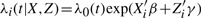

Here, λ_0_(*t*) is an baseline hazard function, *X_i_* is the predictor variable vector coding gene–gene and gene–environment interaction of interest, *Z_i_* is the vector coding for the covariates and *β* and *γ* are the corresponding parameter vectors to *X_i_* and *Z_i_*, respectively. Then, we call *β* the target effects and *γ* the covariate effects.

We propose a new method, called Cox-MDR, using a martingale residual value of the *i*th individual to classify each cell of multi-locus genotype combinations into either high- or low-risk groups. The martingale residual for the *i*th individual is obtained from the null model of no target effects (i.e. *β* = 0) and is specified as follows:

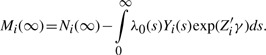

As shown in the equation above, the martingale residual is the difference between the observed and the expected events under the null model with no SNP effects. The sign and the magnitude of the martingale residual would reflect the association of SNPs on the hazard rate. Each individual with a positive martingale residual is classified as a case, whereas one with a negative martingale residual is classified as a control. For each multi-locus genotype combination of SNPs, we calculate the sum of the martingale residuals of those patients who have the corresponding genotype and use it to classify each cell into either high- or low-risk groups. In practice, we assign each cell as high risk if the sum of martingale residuals within that cell is greater than or equal to zero and as low risk otherwise. In Cox-MDR, the martingale residual obtained from the Cox model transforms the continuous survival time into a binary attribute. Hereafter, the MDR's constructive algorithm can be easily applied to the survival time data.

The process of classifying each cell into high- and low-risk groups is summarized as follows:

1. Assume that there are a total of *M* SNPs in the dataset. For a given dataset, fit a reduced Cox model with no SNPs effect, adjusting for covariates. Obtain martingale residuals from the fitted Cox model.
2. For a given *k*-way interaction, select *k* SNPs among *M* SNPs and construct all possible *k*-way contingency tables using these *k* SNPs.
3. For each multi-locus genotype combination defined by the *k* SNPs, calculate the sum of martingale residuals over those individuals who have the corresponding genotype.
4. If the sum of martingale residuals is positive, classify the cell corresponding to this genotype as a high-risk group. Otherwise, classify the cell as a low-risk group.

The Cox-MDR method shares the same reduction strategy as MDR except for replacing the case-control ratios by the sum of the martingale residuals in each cell. For each dataset, balanced accuracy is used to evaluate all possible *k*-way interactions and to identify the best model. The balanced accuracy has been proposed by Velez *et al*. (2007) and is defined as the average of the sensitivity and the specificity as follows:
(1)
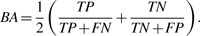

Here, *TP* denotes the true positives, *TN* true negatives, *FP* false positives and *FN* false negatives, respectively. Then, Cox-MDR uses 10-fold cross-validation to determine the best model similarly as implemented in MDR.

As mentioned in Lou *et al.* (2007), the validity of the GMDR method depends on the availability of an appropriate statistic that can provide a measure of the association between the putative factors and the phenotype. Since the martingale residual reflects the unexplained part beyond what is explained by the adjusted covariates with excluding the genetic factors, we can evaluate whether genetic factors have an independent association with the survival time using this martingale residual. In fact, the martingale residual of a Cox model can be easily obtained from a statistical package such as SAS or the R statistical language. The threshold of classification is taken as zero because the expectation of the martingale residual is zero and a positive martingale residual implies that there are more events observed than expected under the model of no SNP effect. In addition, the best combination of genotypes is selected by the balanced accuracy defined in Equation (1) of the MDR procedure.

## 3 SIMULATION RESULTS

Through simulation studies, we compare the power of Cox-MDR with those of Surv-MDR and Cox regression model without and with adjusting for covariates. For comparison, the simulation setting is constructed similar to that of Gui *et al*. (2010).

We consider 2 disease-causal SNPs among 10 unlinked diallelic loci with the assumption of Hardy–Weinberg equilibrium and linkage equilibrium. For the covariate adjustment, we consider only one covariate which is associated with the survival time but has no interactions with any SNPs. We generate simulation datasets from different penetrance functions, which define a probabilistic relationship between a status of high- or low-risk groups and SNPs. We consider eight different combinations of two different minor allele frequencies of (0.2, 0.4) and the four different heritability of (0.1, 0.2, 0.3, 0.4). For each of the 8 heritability-allele frequency combinations, a total of 5 models were generated, which yields 40 epistatic models with various penetrance functions, as described by Velez *et al*. (2007).

Let *f_ik_* be an element from the *i*th row and the *k*th column of a penetrance function. Assuming that SNP1 and SNP2 are the two disease-causal SNPs, we have the following penetrance function:

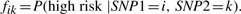

We generate 200 high-risk patients and 200 low-risk patients from each of the 40 penetrance models to create one simulated dataset. For each dataset, we implement 10-fold cross-validation and repeat this procedure 10 times to reduce the fluctuations due to chance divisions of the data. As a result, we generate 100 datasets for each model. We simulate the survival time from a Cox model specified as follows:

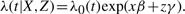

Here, *x* is an indicator variable with value 1 for the high-risk group and 0 for the low-risk group, and we set *β* = 1, *γ* = 0.0, 1.0, 2.0 and *z* is an adjusting covariate generated from *N*(0,0.5). In addition, the baseline hazard function follows a Weibull distribution with the shape parameter of 5 and the scale parameter of 2, and the censoring time is generated from a uniform distribution, *U*(0,4).

For the power comparison, we ran Surv-MDR and Cox-MDR on 100 simulated datasets for each of 40 models, including 2 disease-causal SNPs, and we selected the best model over all possible two-way interaction models without and with adjustment of covariates, respectively. The power of Cox-MDR is defined in the same manner as that of Surv-MDR in Gui *et al*. (2011), in which the power is estimated as the percentage of times Surv-MDR correctly chooses the 2 disease-causal SNPs as the best model out of each set of 100 datasets for each model. In addition, we ran a Cox regression model with each single SNP and estimated the power as the percentage of times that both the two disease-causal SNPs had univariate *P*-value *<* 0.05. Tables 1, 2 and 3 show the power of Surv-MDR, Cox-MDR and Cox-regression model without and with adjustment of covariates when the covariate effect is *γ*= 0.0, 1.0, 2.0, respectively. In Tables 1, 2 and 3, the power is the average of all powers across the same combinations of heritability and minor allele frequencies. Since the Surv-MDR method cannot allow the adjustment of covariate, the power of Surv-MDR is the same regardless of adjustment of covariate.

**Table 1.** Power comparison of Cox-MDR with Surv-MDR and Cox regression model on 40 epitasis models when there is no covariate effect (*γ* = 0.0)

MAF^*^	Heritability	Surv-MDR	Cox-MDR	Cox-regression
0.2	0.1	0.108	0.212	0.066
0.2	0.2	0.266	0.486	0.266
0.2	0.3	0.408	0.678	0.612
0.2	0.4	0.530	0.784	0.806
0.4	0.1	0.170	0.130	0.032
0.4	0.2	0.594	0.354	0.266
0.4	0.3	0.748	0.654	0.500
0.4	0.4	0.920	0.776	0.794

^*^MAF: Minor allele frequency.

**Table 2.** Power comparison of Cox-MDR with Surv-MDR and Cox regression model on 40 epitasis models, with and without adjusting covariates when the effect of covariate is γ= 1.0

		Without adjustment			With adjustment	
MAF^*^	Heritability	Surv-MDR	Cox-MDR	Cox-regression	Cox-MDR	Cox-regression
0.2	0.1	0.058	0.144	0.024	0.202	0.052
0.2	0.2	0.132	0.314	0.092	0.510	0.254
0.2	0.3	0.250	0.474	0.252	0.642	0.580
0.2	0.4	0.384	0.612	0.478	0.812	0.838
0.4	0.1	0.126	0.110	0.016	0.150	0.038
0.4	0.2	0.372	0.224	0.132	0.356	0.302
0.4	0.3	0.560	0.458	0.248	0.664	0.500
0.4	0.4	0.746	0.544	0.446	0.760	0.806

^*^MAF: Minor allele frequency.

**Table 3.** Power comparison of Cox-MDR with Surv-MDR and Cox regression model on 40 epitasis models with and without adjusting covariates when the effect of covariate is γ= 2.0

		Without adjustment			With adjustment	
MAF*	Heritability	Surv-MDR	Cox-MDR	Cox-regression	Cox-MDR	Cox-regression
0.2	0.1	0.038	0.084	0.008	0.196	0.060
0.2	0.2	0.066	0.180	0.032	0.502	0.300
0.2	0.3	0.114	0.244	0.066	0.648	0.600
0.2	0.4	0.124	0.342	0.090	0.798	0.858
0.4	0.1	0.054	0.064	0.006	0.156	0.040
0.4	0.2	0.154	0.082	0.034	0.374	0.312
0.4	0.3	0.250	0.202	0.056	0.658	0.510
0.4	0.4	0.388	0.280	0.128	0.764	0.786

^*^MAF: Minor allele frequency.

For type I error, we also evaluate the performance of Cox-MDR when there is no SNP effect on the survival time. In other words, we check whether the type I error is well preserved under the null hypothesis. To do this, we select randomly 20 datasets from each of 40 models and remove the 2 disease-causal SNPs, which create a total of 800 null datasets. We ran Cox-MDR on 800 datasets and estimated the percentages of times that Cox-MDR included the 2 disease-causal SNPs in the chosen model out of 800 datasets. From the simulation result, the type I error is estimated as 0.02, which is smaller than the nominal level of 0.05. When we tried to select randomly 100 datasets from each of 40 models, which is a total of 4000 null datasets, the type I error varied from 0.014 and 0.040, which is also consistently smaller than 0.05.

As shown in Table 1, the power trend varies over the combinations of minor allele frequency and heritability. The power of three methods steadily increases as the heritability increases from 0.1 to 0.4 though the degree of increase is different over three methods depending on the minor allele frequency. Under the minor allele frequency of 0.2, the power of Cox-MDR is better than those of Cox regression model and Surv-MDR when the heritability is up to 0.2. However, when the heritability is 0.3 and 0.4, the power of Cox-MDR is similar to that of Cox regression model but higher than that of Surv-MDR. However, under the minor allele frequency of 0.4, the power of Surv-MDR is substantially higher than those of Cox-MDR and Cox regression model.

On the other hand, as shown in Tables 2 and 3, when the covariate is not adjusted for, the power of all methods is severely reduced. For example, when the minor allele frequency is 0.4 and the heritability is 0.4, Surv-MDR, Cox-MDR and Cox regression model have the maximum power of 0.388, 0.280 and 0.128, respectively in Table 3 (*γ*=2.0) whereas the corresponding powers are 0.746, 0.544 and 0.446 in Table 2 (*γ*=1.0), respectively. This implies that the power of all methods decreases substantially when the effect of covariate is large and it is not adjusted for. It is noted that there is a similar trend of power when the effect of covariate increases from 2.0 to 3.0 (data not shown). However, the deteriorating power is recovered by adjusting for covariate as shown in Tables 2 and 3. This rationale sounds reasonable because the power of Cox-MDR and Cox regression model increases greatly after adjusting for covariate. As a result, the adjustment of covariate is critically important to detect gene–gene interaction, especially when the effect size of covariate is large. However, Surv-MDR cannot adjust for the covariate and has low power when the covariate is strongly associated with the survival time. On the other hand, Cox-MDR consistently keeps reasonable power while adjusting for covariate even when the effect of covariate is large. Cox regression model also maintains the moderate power like Cox-MDR.

In summary, the simulation results show that Surv-MDR has a good power only when the minor allele frequency is 0.4 and heritability is more than 0.3. Since Surv-MDR cannot adjust for covariate, there is no gains in power by adjusting for covariate and has worse power when the effect size of covariate is larger. On the other hand, Cox-MDR has reasonable power across all combinations of minor allele frequency and heritability and gains substantial power by adjusting for covariate. Cox regression model also has comparable power with Cox-MDR though it is more sensitive to low heritability. This implies that it is very important to adjust for covariates when some of confounding factors are associated with the survival time in detecting significant gene–gene interactions. The availability of covariate adjustment is an important advantage of Cox-MDR and Cox regression model over Surv-MDR.

## 4 REAL EXAMPLE

We analyze a real example of leukemia patient data to illustrate the procedure of Cox-MDR and compare it with Surv-MDR. The data consist of 97 acute myeloid leukemia (AML) patients with demographic and clinical variables and 139 SNPs information. Among those, 40 patients were dead and 57 patients were alive until the termination of study. We consider two variables of age and sex as adjusting covariates in comparing the power of Cox-MDR and Surv-MDR. Likewise the simulation results, we implement Cox-MDR with and without adjusting for covariates up to two-way interactions whereas Surv-MDR is implemented without adjustment of covariate.

First, we ran Surv-MDR and Cox-MDR with all of 139 SNPs for one- and two-way models and the effects of age and sex were adjusted for Cox-MDR. Since the available sample size is only 97 and censoring is heavy, 10-fold cross validation provides too small test set to evaluate the best model. Instead, we implemented 2-fold cross validation with a replication of 100 times and listed the top 3 one- and two-way models in the first column of Tables 4 and 5, respectively. Secondly, we fitted a univariate Cox model with each SNP adjusting for age and sex and listed 21 SNPs that have *P*-value *<* 0.05 in Table 6. Since the MDR method is known to be useful to detect the epistatic models, we removed these 21 SNPs from the dataset and re-ran Surv-MDR and Cox-MDR without SNPs having strong main effects likewise the procedure of Gui *et al*. (2010). The top three one- and two-way models from these results were listed in the right hand side in Tables 4 and 5, respectively. In addition, we displayed *P*-value obtained from the Cox regression model and the permutation *P*-value.

**Table 4.** Top three models identified by Surv-MDR with main effect and without main effect

With all 139 SNPs						With 118 SNPs after removing 21 SNPs having strong main effect					
Models	TSSC	TSSC	Coeff.	*P*	P^*^	Models	TRSC	TSSC	Coeff.	*P*	P^*^
One-way						One-way					
NT5C3 rs12155477	25.435	25.595	−0.045	0.844	**0.00936**	NT5C3 rs12155477	25.607	25.595	−0.045	0.844	**0.00936**
SLC29A1 rs7753792	20.257	16.398	2.326	**0.003**	0.05509	DCK rs4694362	11.238	11.291	0.429	0.145	0.07387
DCTD rs13139377	13.951	13.659	0.767	**0.003**	0.28089	TYMS rs1004474	10.825	10.730	0.156	0.487	0.04114

Two-way						Two-way					
NT5C3 rs12155477 and	42.880	43.174	−0.083	0.839	**0.01828**	DCK rs4694362 and	38.108	37.876	0.556	0.234	**0.00189**
DCTD rs13114435						NT5C3 rs12155477					
NT5C3 rs12155477 and	42.732	43.143	−0.101	0.804	**0.00856**	NT5C3 rs12155477 and	37.328	37.464	0.100	0.722	**0.00152**
DCTD rs6552621						TYMS rs2847153					
NT5C3 rs12155477 and	42.662	43.038	−0.088	0.830	**0.00816**	NT5C3 rs12155477 and	36.978	37.465	3.317	**0.012**	**0.00873**
DCTD rs17331744						NT5C3 rs7776847					

TRBA: Training balanced accuracy; TSBA: Testing balanced accuracy; Coeff.: the estimated effect size of the corresponding SNP effect; *P*: *P*-value of main and two-way interactions in the Cox regression model; P*: permutation *P*-value of main and two-way interaction effects.

**Table 5.** Top three models identified by Cox-MDR with main effect and without main effect

With all 139 SNPs						With 118 SNPs after removing 21 SNPs having strong main effect					
Models	TRBA	TSBA	Coeff.	*P*	P^*^	Models	TRBA	TSBA	Coeff.	*P*	P^*^
One-way						One-way					
TYMS rs1004474	0.665	0.665	0.156	0.487	**0.00037**	TYMS rs1004474	0.665	0.665	0.156	0.487	**0.00037**
TYMS rs2847153	0.633	0.633	0.206	0.324	**0.00194**	TYMS rs2847153	0.633	0.633	0.206	0.324	**0.00194**
CDA rs10799647	0.629	0.630	−0.723	0.076	**0.00001**	CDA rs10799647	0.629	0.630	−0.723	0.076	**0.00001**

Two-way						Two-way					
CDA rs12404655 and	0.719	0.712	−1.125	0.098	**0.00001**	CDA rs12404655 and	0.719	0.712	−1.125	0.098	**0.00001**
TYMS rs1004474						TYMS rs1004474					
CDA rs532545 and	0.705	0.704	−0.216	0.591	**0.00973**	CDA rs532545 and	0.705	0.704	−0.216	0.591	**0.03013**
TYMS rs2847153						TYMS rs2847153					
CDA rs10916824 and	0.713	0.704	−1.211	0.117	**0.00138**	MTHER rs9651118 and	0.721	0.703	−0.714	**0.038**	**0.00035**
TYMS rs1004474						TYMS rs1004474					

TRBA: Training balanced accuracy; TSBA: Testing balanced accuracy; Coeff.: the estimated effect size of the corresponding SNP effect; *P*: *P*-value of main and two-way interactions in the Cox regression model; P*: permutation *P*-value of main and two-way interaction effects.

**Table 6.** 21 SNPs with main effect under *P*-value (<0.05) from a univariate Cox model adjusting for age and sex

SNP	Coeff.	*P*-value	FDR^*^
SLC29A1 rs7753792	2.3255	0.0029	0.1313
DCTD rs13139377	0.7668	0.0033	0.1313
DCTD rs17331744	0.7435	0.0118	0.1313
DCTD rs7663494	0.7435	0.0118	0.1313
DCTD rs3886768	0.7435	0.0118	0.1313
DCTD rs13148414	0.7435	0.0118	0.1313
DCTD rs17331968	0.7435	0.0118	0.1313
DCTD rs10520543	0.7435	0.0118	0.1313
DCTD rs9990999	0.6316	0.0128	0.1313
DCTC rs13116494	0.6316	0.0128	0.1313
DCTD rs13116598	0.6316	0.0128	0.1313
DCTD rs3811810	1.1114	0.0133	0.1313
DCTD rs13114435	0.7336	0.0134	0.1313
DCTD rs6552621	0.7224	0.0138	0.1313
DCTD rs7688234	0.6226	0.0146	0.1313
DCTD rs13101260	0.6064	0.0151	0.1313
SLC29A1 rs1057985	−0.5770	0.0228	0.1866
DCTD rs10009825	0.6555	0.0264	0.2038
SLC29A1 rs507964	−0.5195	0.0380	0.2780
CDA rs10916824	−1.0402	0.0447	0.3093
DCTD rs17272827	0.5684	0.0467	0.3093

^*^False discovery rate

As can be seen in Table 4, for Surv-MDR, the results with all139 SNPs are quite different from those with 118 SNPs after removing 21 SNPs having significant main effect because the 2 SNPs, SLC29AL rs7753792 and DCTD rs13139377, were detected to be top 3 main effects but these 2 SNPs did not have significant permeation *P*-values in detecting the two-way interactions when all 139 SNPs were considered. However, when 118 SNPs were considered, NT5C3 rs12155477 was selected as the top one-way model and also appeared in detecting two-way models.

On the other hand as shown in Table 5, for Cox-MDR, the results with all 139 SNPs are almost the same as those with 118 SNPs except for one- or two-way models, since the top 3 SNPs were not detected significantly by the single SNP approach using a Cox model. Among those, TYMS rs1004474 and TYMS rs2847153 also appear in top three two-way models. It is noted from Tables 4 and 5 that no pairs were commonly detected as two-way models by both Surv-MDR and Cox-MDR.

**Fig. 1.**
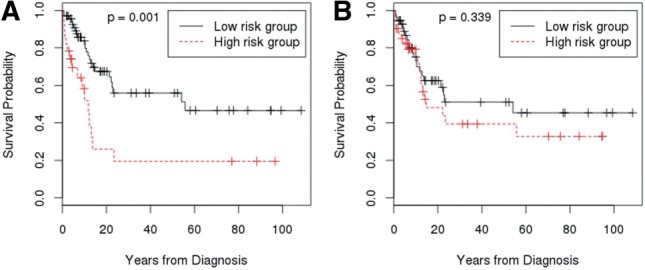
AML survival curves for the high-risk versus low-risk groups by the attribute of SNP pairs selected by Surv-MDR. (**A**) NT5C3 rs12155477 and DCTD rs13114435 (**B**) NT5C3 rs12155477 and NT5C3 rs7776847

**Fig. 2.**
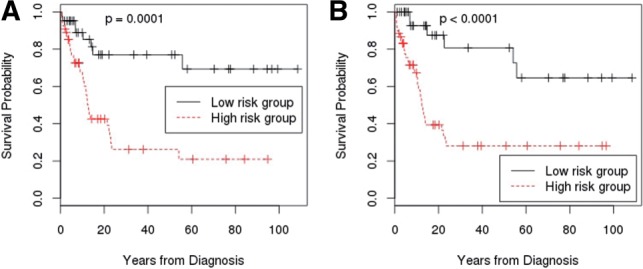
AML survival curves for the high-risk versus low-risk groups by the attribute of SNP pairs selected by Cox-MDR. (**A**) CDA rs12404655 and TYMS rs1004474 (**B**) MTHER rs9651118 and TYMS rs1004474

In order to compare the performance of Surv-MDR and Cox-MDR, we plot the survival curves for the high- versus low-risk groups by the attribute of SNPs pairs selected as two-way models in Figures 1 and 2. In Figure 1, two plots display the survival curves for high- and low-risk groups defined by the attributes of two-way models by Surv-MDR with *P*-values of the log-rank test for the equality of two survival curves. The Surv-MDR attribute in Figure 1A is defined by NT5C3 rs12155477 and DCTD rs13114435 and significantly separate two survival curves with *P* = 0.001. However, the Surv-MDR attribute of NT5C3 rs12155477 and NT5C3 rs7776847 do not separate two survival curves clearly as shown in Figure 1B. The two survival curves cross in the early time and yield no significant log-rank test result with *P* = 0.339. The other attributes also separate two survival curves significantly (data not shown).

Similarly, we plot the survival curves for high- and low-risk groups by the attributes of two-way models defined by Cox-MDR in Figure 2. Two plots of Figure 2A and B show the significant separation of the survival curves for high- and low-risk groups with the significant *P*-values for the log-rank test.

It is noted that the SNPs defined as attributes of Surv-MDR and Cox-MDR are not included in the list of Table 6 because these cannot be detected to be significantly associated with the survival time for the single SNP approach with a Cox model. As shown in Tables 4 and 5, the univariate Cox regression *P*-values of NC5C3 rs12155477, TYMS rs1004474 and TYMS rs2847153 are 0.844, 0.487 and 0.324, respectively. However, Surv-MDR and Cox-MDR select these SNPs in one-way and two-way models and the combinations of these SNPs make subjects separate high- and low-risk groups significantly. This implies that there may be gene–gene interactions associated with the survival time which cannot be detected by ordinary approach using a Cox model.

## 5 DISCUSSION

To identify the complexity of gene–gene and/or gene–environment interactions on common diseases, many plausible approaches have been developed by extending existing methods into a more general framework. In this article, we propose the Cox-MDR method by extending the main idea of GMDR to the survival phenotype. Cox-MDR uses the martingale residual of the Cox regression model as a score to classify multi-loci genotype combinations into high-and low-risk groups. Since the martingale residual reflects the unexplained part beyond what is explained by the adjusted covariates excluding the genetic factors, we can evaluate whether genetic factors have an independent association with the survival time using the martingale residual.

Through the simulation study, we compared the performance of Cox-MDR to those of a Cox regression model and Surv-MDR. All of the three methods showed the common trend that the power increases steadily as heritability increases from 0.1 to 0.4 although the degree of increase varies depending on the minor allele frequency and the effect size of covariate. When the minor allele frequency is 0.2, Cox-MDR and Cox regression model have higher power than Surv-MDR but Surv-MDR has higher power than Cox-MDR and Cox regression model when the minor allele frequency is 0.4 regardless of the effect size of covariate.

It is noted that the power of three methods decreases substantially as the effect size of covariate increases from 1.0 to 2.0 as shown in Tables 2 and 3. It might be that the association of gene–gene interaction on the survival time could be confounded by the unadjusted covariates. This can be seen that after adjusting for covariate, Cox-MDR and Cox regression model recover the reasonable power regardless of the effect size of covariate, whereas Surv-MDR has worse power as the effect size of covariate becomes larger. This implies that the adjustment of covariates is very important to detect multi-loci genetic effects on the survival time when the genetic effect on the survival time is commonly confounded by demographic or clinical covariates, such as age, sex, race and blood pressure. Both Cox-MDR and Cox regression model have great advantages over Surv-MDR due to feasibility of adjusting for covariate.

From the result of real data analysis, it is noted that the top ranked SNP pairs identified by Surv-MDR and Cox-MDR separate the survival curves for the high- and low-risk groups significantly except for one case. Each of these SNPs has no significant main effects in the Cox model but the gene–gene interaction effect defined by their pairs has substantial impact on the separation of two survival cures between high- and low-risk groups.

Comparing Cox-MDR and Surv-MDR, Surv-MDR is a non-parametric approach based on the log-rank test statistic whereas Cox-MDR is a semi-parametric approach based on the martingale residual of a Cox model. When Surv-MDR calculates a log-rank test statistic to pool multiple combinations of genotypes into two-level attributes, each log-rank test statistic is comparing the survival time between samples with and without the genotype combination. For example, we consider two-way interactions between SNP1 with allele A and *a* and SNP2 with B and *b*, in which there are nine possible genotypes from the combinations of SNP1 and SNP2. For the cell of genotype (AA, BB), Surv-MDR computes a log-rank test statistic comparing the survival time between samples with the genotype (AA, BB) and without this genotype. However, for the cell of genotype (A*a*, BB), those patients who have the genotype of (AA, BB) are also used for computing the log-rank test statistics as the alternative group for comparing the survival time between samples with a genotype of (A*a*, BB) and without this genotype combination. In this way, each individual contributes several times to compute the log-rank test statistic as either one of the samples with or without the genotype of interest. This overlapping usage may cause distinctions between high- and low-risk groups to become contaminated, which yield rather low power. However, the martingale residual of each individual is taken into account once to discriminate each cell into high- or low-risk groups, such as the case-control ratios in MDR.

The execution time of our Cox-MDR has linear relationship with sample size and combinations of SNPs. We expect that Cox-MDR can evaluate 5 × 10^9^ combinations (e.g. pairwise interactions between 100k SNPs) with 1000 samples and 10 cross validation in ~6.4 days on a workstation with Intel Xeon 2.4GHz CPU and 12G RAM. If Cox-MDR is extended to parallel computing system, our Cox-MDR can be feasible to analyze pairwise interactions for GWAS.

In conclusion, Surv-MDR needs more intensive computations for a large number of SNPs and has a big weakness with which there is no way to adjust for covariates whereas, Cox-MDR requires less intensive computations by using the martingale residual score and has a great advantage of being able to adjust for covariate. Moreover, the Cox-MDR method could be extended to other types of high-dimensional data such as copy number variation (CNV) and next generation sequencing (NGS) data.

For the next research topic, we plan to work on using the standardized residual for the parametric regression models in the frame of GMDR. Our key idea can be extended to the parametric regression models with various error distributions such as Weibull, log-normal and logistic distributions.

*Funding*: This work was supported by the National Research Foundation of Korea (NRF) grant funded by the Ministry of Education, Science and Technology of Korea (MEST) (20110507 and 2012R1A3A2026438).

*Conflict of Interest:* none declared.
